# Cantilever-Droplet-Based Sensing of Magnetic Particle Concentrations in Liquids

**DOI:** 10.3390/s19214758

**Published:** 2019-11-01

**Authors:** Wilson Ombati Nyang’au, Andi Setiono, Maik Bertke, Harald Bosse, Erwin Peiner

**Affiliations:** 1 Institute of Semiconductor Technology (IHT) and Laboratory of Emerging Nanometrology (LENA), Technische Universität Braunschweig, 38106 Braunschweig, Germanye.peiner@tu-braunschweig.de (E.P.); 2 Department of Metrology, Kenya Bureau of Standards (KEBS), Nairobi 00200, Kenya; 3 Research Center for Physics, Indonesian Institute of Sciences (LIPI), Kawasan Puspiptek Serpong, 15314 Tangerang Selatan, Indonesia; 4 Precision Engineering Division, Physikalisch-Technische Bundesanstalt (PTB), 38116 Braunschweig, Germany

**Keywords:** microcantilever, resonant frequency, magnetic polystyrene particles, droplet, adsorption, mass, concentration

## Abstract

Cantilever-based sensors have attracted considerable attention in the recent past due to their enormous and endless potential and possibilities coupled with their dynamic and unprecedented sensitivity in sensing applications. In this paper, we present a technique that involves depositing and vaporizing (at ambient conditions) a particle-laden water droplet onto a defined sensing area on in-house fabricated and commercial-based silicon microcantilever sensors. This process entailed the optimization of dispensing pressure and time to generate and realize a small water droplet volume (*V*_d_ = 49.7 ± 1.9 pL). Moreover, we monitored the water evaporation trends on the sensing surface and observed total evaporation time per droplet of 39.0 ± 1.8 s against a theoretically determined value of about 37.14 s. By using monodispersed particles in water, i.e., magnetic polystyrene particles (MPS) and polymethyl methacrylate (PMMA), and adsorbing them on a dynamic cantilever sensor, the mass and number of these particles were measured and determined comparatively using resonant frequency response measurements and SEM particle count analysis, respectively. As a result, we observed and reported monolayer particles assembled on the sensor with the lowest MPS particles count of about 19 ± 2.

## 1. Introduction

With the recent advancement in micro- and nanofabrication technologies, cantilever-based sensors have increasingly become versatile tools for various sensing applications due to their fascinating intrinsic flexibility and robustness. They have widely been embraced and applied in many scientific fields ranging from physical and chemical sensing, material characterization, magnetometry, explosive detection, to biological disease diagnosis [1,2,3,4]. For instance, they have immensely and significantly contributed to the biopharmaceutical development, manufacture and quality assurance, in which the titer of virus is related to the viral dosage and resultant performance of the vaccine [5]. Moreover, these sensors play a critical role in protein aggregation studies, differentiation and concentration measurement of sub-visible protein aggregates and silicone oil droplets in pre-filled syringe aggregation studies. In drug delivery [6,7], cantilever-based sensors facilitate the measurement of the concentration and particle size of the delivery vector, which often influence the biological response to the drug. Similarly, in disease diagnostics, they have been deployed in determining the concentration of specific exosomes and microvesicles [8], which may be indicative of the onset of a disease. They are also widely used in nanoparticle toxicology studies [2] to determine the concentration of particles within a biological or ecological environment, which essentially influences the biological response to the nanoparticles from a toxicity perspective, etc.

In the measurement of particle concentration, for instance, some various measurement technologies such as dynamic light scattering, nanoparticle tracking analysis, and resonant mass sensing can be deployed. These technologies provide particle concentration measurement based on particle fluorescence, particle size analysis and ensemble particle zeta potential measurement, and particle mass measurements, respectively. For the latter, differential stress at the surface to the beam of the cantilever causes bending or deflection of the cantilever. Most equipment for determining small masses, e.g., mass spectrometers [9] and suspended microchannel resonators [10], often utilize complicated technology and are quite expensive. On the other hand, the scope of application of Quartz Crystal Microbalances (QCMs) has been extended beyond gaseous to liquid media (e.g., in the study of the environment, nanoparticles, etc.). However, the interaction of the QCM surface with the liquid often results in the formation of soft or viscoelastic films. Besides, spurious factors (e.g., stress, pressure, etc.) exist [11], which potentially dampen the vibrations. This has subsequently necessitated the recent deployment of QCM with dissipation monitoring [12] to measure both the resonant-frequency change (Δ*f*) and energy loss (measured as dissipation change).

In providing a low-cost measurement approach, dipping a cantilever sensor into a magnetic particle solution has also recently been devised and implemented [13]. However, this particle sampling and measurement approach results in low quality factor (*Q* ~ 180) due to severe damping and often yields randomized adsorption of particles on the whole cantilever surface. Furthermore, uniformity in particle adsorption or distribution over the sensing region is never guaranteed. In our study, we explored and presented an alternative droplet-based approach that can be utilized to precisely deposit particles on a specified sensing area on a cantilever for particle concentration or mass sensing applications. We successfully demonstrated localized deposition (utilizing liquid dispensing) of monodispersed magnetic polystyrene (MPS) particles onto a piezoresistive silicon cantilever sensors. Furthermore, we discussed some uncertainty parameters that influenced our measurements.

Through an optimized liquid-dispensing mechanism, we sub-sampled and loaded small water droplets with and without particles onto in-house fabricated cantilever sensors. Additionally, as a self-validating process, particle-laden droplets were similarly deposited on surface-treated silicon substrates and upon solvent evaporation, particle count estimations were performed using scanning electron microscopy (SEM). We also monitored and determined the droplet evaporation time (*t*_ev_) and trends thereof for water droplets of known volume (*V*_d_). The particles used herein included magnetic polystyrene (MPS) from Micromod GmbH, and polymethylmethacrylate (PMMA) from Sigma-Aldrich Inc. It is worth noting that the two test particles have comparable (nominal) diameters and densities, but contrasting magnetic properties, with MPS and PMMA being magnetic and non-magnetic, respectively. However, similar to silicon, PMMA has magnetic susceptibility of less than 1 and is thus diamagnetic in nature. The essence of using these two particle types was to compare the results and assess the performance of our measurement approach.

Magnetic polymer particles (such as MPS) are usually synthesized by embedding magnetic nanoparticles (e.g., from pure metals such as Fe, Co and Ni or their oxides) into a non-magnetic spherical (polymer) matrix [14]. Magnetic beads come in different compositions, sizes and shapes. These parameters determine their chemical and physical properties. These particles can be coupled with ligands that target specific molecules. This consequently makes them highly versatile and allows their widespread use in industrial and biomedical applications. For instance, in biomedicine, they are often useful in diagnosis and therapy, e.g., sorting and purification of cells, proteins and nucleic acids, as contrast reagents in magnetic resonance imaging (MRI), targeted drug delivery, diagnostics and targeted destruction of tumors by intercellular hyperthermia [15,16,17]. In the industry, these particles similarly play a critical role, e.g., as inorganic solar cell constituents, chemically inert additives, cosmetic UV protection and color filters for LCDs [18], optimization of bioengineering processes especially in the oil industry [14], high-density data storage [19,20], etc. In environmental monitoring, magnetic nanoparticles can also be used directly for pollutant removal [21], e.g., from contaminated water resources. They for instance facilitate the removal of mercury ions in aqueous solutions by sensing, sorting and quantifying them using a nanoparticle-based system with optical and electrochemical detection regimes [22].

## 2. Materials and Methods

### 2.1. Magnetic Polystyrene Particles

In this study, we used magnetic polystyrene (MPS) beads of nominal diameter ~2 μm and density 1.1 gcm^−3^, from micromod Partikeltechnologie GmbH (D-18119 Rostock, Germany). They are basically monodispersed particles consisting of magnetite (Fe_3_O_4_) around an organic matrix of a styrene-maleic acid (SMA) copolymer, which are finally encapsulated with a polymer (polystyrene) layer [14] Hence, MPS exhibits a superparamagnetic behavior. The surface of the particles, herein used, is not chemically functionalized.

Originally, monodispersed MPS particle solution was obtained in an aqueous suspension of 50 mg/mL concentration and thoroughly mixed (by sonication) prior to use or dilution. The solution was otherwise diluted with deionized water to tune the concentration levels, i.e., into approximately 20, 10, 1 and 0.1 mg/mL. This was the main variable, besides the droplet size, through which the mass Δ*m* or the number *N*_p_ of particles was varied for every particle solution dispensed on the target substrate or sensing surfaces.

### 2.2. Silicon Substrates Preparation and Surface Treatment

The silicon bulk substrates (~15 × 15 mm^2^) used in this study were cut from an *n*-type Si (100) wafer (Siegert Wafer GmbH, Aachen, Germany). These wafers had resistivity and thickness of 1–10 Ωcm and 275 ± 15 µm, respectively. After cleaving, the substrates were individually cleaned by: (a) boiling (5 min) in an oxidant solution containing a mixture of sulfuric acid (H_2_SO_4_, 96%) and hydrogen peroxide (H_2_O_2_, 30%) in a volume ratio of 1:1; (b) immersing in a water bath (for 5 min); (c) rinsing with deionized water; and (d) blow drying with nitrogen gas. Such a cleaning process was necessary in removing organic contaminants (e.g., dust particles, grease, silica gel, etc.) and ionic or metal contaminants from the silicon surface, prior to use or surface treatment.

Upon cleaning, some pre-selected bulk substrates were then dipped in HF solution (6–7%) for about 5 s (to etch the native oxide from silicon surface). Immediately thereafter, some set of samples were treated with O_2_ plasma (100-E, Technics Plasma GmbH) for 2 min at RF power of 220 W, while others were treated with PSS/PDDA/PSS [23]. This was meant to yield different wettabilities (i.e., surface properties). Consequently, water contact angle *θ* was measured for each set of substrates: as-cleaned and after O_2_ plasma treatment.

Measurement of contact angle was carried out by the sessile-drop method using a dispensing system integrated with an imaging system which comprises of a light source, a collimating mask, an adjustable stage, and a digital microscope camera (Mz-902, Oowl Tech Ltd., Hong Kong, China). The latter was helpful in visualizing and capturing the water droplet on each silicon substrate. Careful attention was taken to generate a homogeneous background behind the drop. For this reason, we positioned a diffuser between the lamp and the water droplet. The height of the camera was also adjusted so that the actual drop and its reflection on the silicon surface could clearly be observed. Consequently, the intersection point at the solid–liquid–air interfaces would precisely be determined. The internal nominal diameter of the dispensing tip (Nordson EFD Inc., East Providence, RI, USA) was ~0.10 mm, and a low pressure (~0.06 bar) was applied to dispense a droplet at room temperature (21.8 ± 0.5 °C).

**Figure 1:**
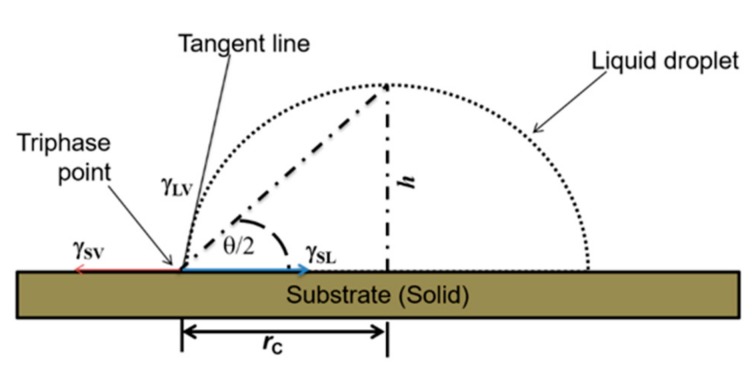
Schematic of contact angle *θ* measurement by the half-angle method.

In determining the contact angle, the profile of the drop image was extracted by an image-fitting in ImageJ [24] using the Low Bond Axisymmetric Drop Shape Analysis (LBADSA) method [25]. Primarily, by observing the shape of such a drop profile, the contact angle *θ* can be measured, in which case *θ* is the angle formed between the drop tangent and the solid surface at the tri-phase contact point of a simple spherical droplet, as depicted in Figure 1. This approach allows for calculation of *θ* from sessile drop images based on the Young–Laplacian equation (Equation (1)) for spherical approximation for half-angle method, in which *r*_c_ and *h* denote the contact radius and the height of the drop above the solid surface, respectively.

(1) $$\frac{\theta }{2}{=\tan}^{-1}\left(\frac{h}{r_{c}}\right)$$

Furthermore, the angle *θ* depends on the surface tensions for solid–liquid (*γ*_sl_), solid–air (*γ*_sv_), and liquid–air (*γ*_lv_) interfaces. For small drops (of liquid density *ρ*), surface tension dominates over gravity, *g* ≈ 9.81 ms^−2^. Consequently, a drop tends to form a spherical-cap shape to minimize total free surface energy thereby resulting into a capillary length, or capillary constant *L*_c_, given as:

(2) $$L_{c}\;=\frac{\Delta \rho \cdot g}{\gamma_{lv}}.$$

The parameter Δ*ρ* represents the difference in density of the involved phases (i.e., liquid and air). For water (*ρ* = *ρ*_w_ = 1 gcm^−3^, *γ*_lv_ = 0.0728 Nm^−1^) at room temperature and standard pressure conditions, *L*_c_ ≈ 1.35 × 10^−5^ m^−2^. The LBADSA method further considers the pressure gradient across a surface to the drop curvature, and the contact angle is usually approximated based on a perturbation solution of the contour of an axisymmetric sessile drop and assumes gravity as the only external force, and follows Equations (2) and (3). Equation (3) relates the principal radii of curvature (*R*_1_ and *R*_2_) at any point on the drop, the height *h* of the drop, the capillary constant *L*_c_ and the radius of curvature *b* at the apex.

(3) $$h=\;\frac{1}{L_{c}}\left(\frac{1}{R_{1}}+\frac{1}{R_{2}}-\frac{2}{b}\right).$$

In our study, as-cleaned samples yielded *θ* = 47.3° ± 2.7°, whereas contact angle after O_2_ plasma treatment (2 min) was about 9°. Obviously, our surface treatment with O_2_ plasma yielded enhanced hydrophilicity compared to the 24.5–46.5° (for 1–20 min O_2_-RIE plasma treatment duration) reported by Alam et al. [26]. It is worth noting that a short-time lapse between surface treatment and contact angle measurements was observed in our study. Such short time gap does not compromise the results, which can otherwise be a consequence of buildup of static charges. Moreover, for our evaluation of the experimental contact angle, we assumed a negligible effect of gravity and a constant droplet volume. This can therefore be attributed to the measurement of contact angles immediately after O_2_ plasma treatment.

### 2.3. Fabrication of Silicon-Based Piezoresistive Cantilever Mass Sensors

**Figure 2:**
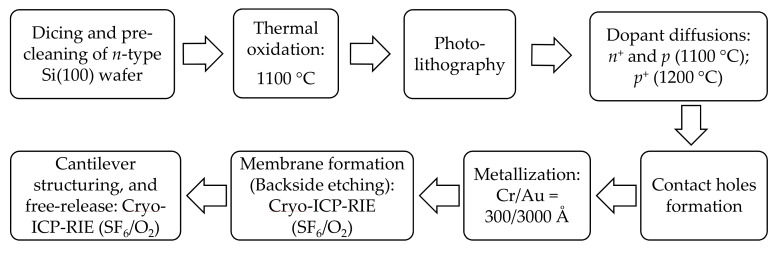
Schematic illustration of the main parameters and processes undertaken during the in-house fabrication of silicon-based cantilever sensors by bulk micromachining.

Fabrication process of our cantilever-based sensors was exclusively done in-house, using *n*-type (100) silicon (as a base material) and conventional bulk micromachining technologies as described by Wasisto et al. [27]. The process mainly entails substrate preparation (i.e., dicing the bulk Si wafer into 30 × 30 mm^2^ pieces and cleaning them accordingly; cf. Section 2.2), thermal oxidation, photolithography, dopant diffusions (phosphorous and boron), etching processes (i.e., HF dip and cryogenic ICP-RIE: SF_6_/O_2_), contact holes formation, and metallization of electrical contacts (Cr/Au = 300/3000 Å) coupled with a lift-off process. The general cantilever fabrication process utilizing bulk micromachining is schematically illustrated in Figure 2.

Fabrication process basically began with the deposition of an isolation layer (SiO_2_) on top of the pre-cleaned silicon substrate at 1100 °C using thermal oxidation process. Hereafter, an intermediary substrate-cleaning process was performed by sonication in acetone and water rinses followed by nitrogen blow-dry. The silica layer (~100 nm) provided an excellent protection during dopant diffusion processes, in which case heating furnace temperature was set and maintained at 1100 °C (*n*^+^- and *p*-diffusions) and at 1200 °C (*p*^+^-diffusion).

Photolithography was then undertaken to pattern and transfer various geometric shapes/structures (e.g., U-shaped Wheatstone bridge piezo resistors, connection lines and contact pads) on a mask to the surface of the silicon substrates. A preliminary surface preparation process involving bubbling of hexamethyldisilazane (HMDS) within an enclosed chamber was executed (at ~115 °C for ~5 min) to enhance hydrophobicity of the substrate. Hereafter, pattern transfer ensued. Firstly, the prepared substrate was held onto a vacuum chunk and a photoresist (AZ 5214) was dispensed onto the silicon surface and spin-coated (e.g., 3500 rpm for 35 s). The sample was then partially evaporated in a soft baking process (110 °C for ~50 s) to improve adhesion and uniformity, and optimize light absorbance characteristics of the photoresist. To transfer the mask image to the resist-coated wafer, both the mask and substrate were mounted onto respective stages/holders on the mask aligner equipment (MBJ4, SÜSS MicroTec AG, Garching, Germany) followed by alignment and UV exposure (for specified time period, e.g., 35 s). To dissolve the soluble areas of the exposed photoresist and realize visible patterns on the wafer, the sample was immersed and gently agitated in a developer chemical solution (AZ 726) for ~60 s and thoroughly rinsed with deionized water. The resulting pattern after resist development would then be inspected (under a light microscope) followed by a hard-baking process (at 100 °C for ~30 min). Subsequently, the hardened-patterned sample was immersed in a buffered hydrofluoric acid for about 10 min to remove the entire SiO_2_ layer or ~5 s to clear the native oxide as desirable.

After forming piezo resistors, contact holes, electrical connections and contact pads, the sample was cryogenically etched (ICP-RIE, with SF_6_/O_2_) from the backside to create a membrane. Lastly, the patterning process (by lithography) was undertaken to form the desired free-ends (i.e., rectangular or triangular beam configurations [28]) of our cantilever sensors. This was then followed by the free-releasing of the cantilever sensors from the sample through a cryogenic dry etching process. The length and width of rectangular cantilevers were 1000 µm and 170 µm, respectively, while the size of the equilateral-shaped free-end triangular cantilever was ~700 µm. The thickness of our sensors was approximately 37.9 ± 0.2 µm, and the mass *m*_0_ of cantilever was 15.01 ± 0.11 µg and 24.65 ± 0.18 µg for rectangular and triangular cantilevers, respectively.

### 2.4. Droplet Dispensing Apparatus

**Figure 3:**
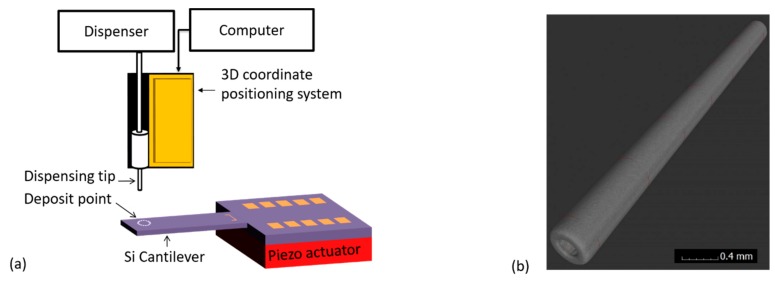
(**a**) Schematics of liquid (and particles) dispensing setup; and (**b**) X-ray computer tomography (xCT) image of the dispensing stainless needle tip.

Water droplet with/without MPS particles was generated from a needle tip (of specified size) by applying specified dispensing pressure over a defined pulse duration. By keeping the size of the needle tip constant, the drop size could reliably be tuned mainly by varying the magnitude of the dispensing pressure and/or time. Through X-ray computer tomography (xCT) measurements (undertaken at PTB), we precisely calibrated and determined the internal and external diameters of our dispensing stainless needle tip (see Figure 3) to be 0.1169 ± 0.004 mm and 0.2357 ± 0.0005 mm, respectively. The latter was also necessary and helpful in calibrating our USB digital camera (Mz-902, Oowltech).

Firstly, each cantilever mass sensor was mounted on top of a piezo actuator within the sampler and carefully aligned in the horizontal plane. At this point, the particles collection target point would be defined (*X*,*Y*), i.e., by specifying a coordinate along the axisymmetric and the vibrating axis of the cantilever. The needle tip would then be mounted into a barrel (containing a thoroughly mixed solution of MPS particles) and aligned directly on top of deposit target point. During the alignment process, a 3D micro-positioning system (with a resolution of 10 μm) was used to carefully move the needle tip to the target point; and with an aid of a camera, the height from the sensor and the tip would carefully be adjusted (*Z*-coordinate). To further enhance the accuracy of positioning the tip on the sensing surface and minimize sensor breakages, a camera was also incorporated in the alignment process. The tip was aligned and adjusted very closely above the sensor target deposit point, in orders of few microns. Finally, the droplet was generated (1500XL, Nordson EFD Inc., Rhode Island,USA) and deposited on the sensor by applying defined dispensing parameters (i.e., air pressure and pulse duration). Typically, minimum dispensing pressure (~60 mbar) was applied and different pulse durations were used. This was desirable in generating a small droplet as much as possible.

### 2.5. Resonant Mass Sensing Instrumentation

**Figure 4:**
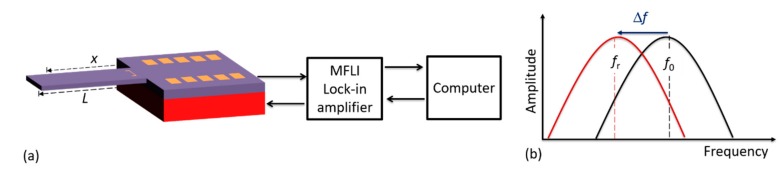
Schematic illustration depicting: (**a**) the resonant frequency measurement setup; and (**b**) the expected resonant frequency shift Δ*f* due to the mass Δ*m* of attached media.

In detecting cantilever bending due to the mass of the droplet or adsorbed particles, different sensing or read-out methods can be deployed, such as piezoelectric, electrodynamic, tunneling, hard contact, optical reflection, interferometric, capacitive, and piezoresistive techniques [29]. The latter mechanism was preferably integrated into our cantilevers during the fabrication process and was realized in the form of a U-shaped Wheatstone bridge by diffusing piezo resistors into the cantilever. To supply voltage (1 V_dc_) and detect the voltage output from the bridge, a lock-in amplifier (MFLI, Zurich Instruments Ltd.) was connected to the cantilever contact pads (as shown in Figure 4). Since our microcantilevers were externally excited with an in-plane piezoelectric stack actuator (P-121.05, from PI Ceramics GmbH, Lederhose, Germany), an excitation voltage (9.9 V_pp_) was similarly supplied to the actuator by the MFLI instrument. The connections to and from the MFLI were accomplished through coaxial cables and SubMiniature version A (SMA) connectors. The required MFLI parameters (e.g., input voltage) and the signal read-out from sensor (e.g., frequency response) were managed using a computer via a control software.

Initially, and for each particle adsorption cycle, the resonant frequencies of the cantilever before (*f*_0_) and after (*f*_r_) load deposition were measured. This was necessary to precisely determine the resonant frequency shift (Δ*f* = *f*_r_ − *f*_0_) due to the loaded mass Δ*m* (i.e., mass of the droplet or adsorbed particles). Consequently, the particle and/or droplet mass was determined at a point *x* from the fixed end of the cantilever beam of length *L*. With the measured resonant frequency shift Δ*f*, the mass of the cantilever *m*_0_ and the mode-shape function *U*(*x*_Δm_), the drop or adsorbate mass Δ*m* can be calculated according to [30]:

(4) $$\Delta m\;=\;\frac{m_{0}}{U^{\;2}{(x}_{\Delta m})}\left(\frac{f_{0}{}^{2}}{f_{r}{}^{2}}-1\right),$$

where $U\left(x_{\Delta m}\right)=\left(\cos\;\lambda \;+\;\cosh\;\lambda \right)\left(\cos(\lambda \frac{x}{L})-\cosh(\lambda \frac{x}{L})\right)+\left(\sin\lambda -\;\sinh\;\lambda \right)\left(\sin(\lambda \frac{x}{L})-\sinh\left(\lambda \frac{x}{L}\right)\right).$ The parameter *λ* represents a modal constant, which is about 1.8751 for the first mode. To begin a new measurement cycle, the adsorbed particles were removed/cleaned from the cantilever surface and *f*_0_ was measured again to average and compare with the initially obtained value.

### 2.6. Cleaning and Removal of Adsorbed Particles from the Cantilever

Whenever particles or analytes are collected on a silicon cantilever for mass detection or otherwise, they often adhere on the sensing surface. For instance, airborne particles such as cigarette smoke sticking on the surface of silicon cantilever can easily be removed by immersing and gentle agitating the cantilever in a glass containing acetone (for a few minutes) [31]. However, after adsorbing liquid-dispersed MPS particles on the silicon cantilever, conventionally much more effort is required to remove and clear these particles from the sensing surface.

**Figure 5:**
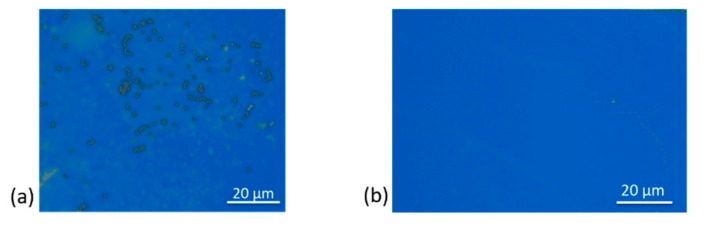
Optical images of a silicon cantilever surface: (**a**) containing adsorbed magnetic polystyrene beads (dark round features); and (**b**) after sonication treatment (in which all particles are completely removed from the surface).

In this work, therefore, regeneration of particle-adsorbed cantilever sensors (Figure 5a) was mainly performed by immersion in acetone and sonication for about 3–5 min. With this approach, the particles were detached and cleared from the surface (Figure 5b). Since cleaning the fragile silicon cantilevers by sonication is a risky process, often bound to break and destroy the sensors, we clipped each particle-laden cantilever on the flat surface of a metallic mounting before immersing it in a glass containing acetone. This mechanism was successfully implemented and resulted in nearly 100% particle removal efficiency and alleviated the risk of damaging the cantilevers.

## 3. Droplet Size Optimization and Evaporation

### 3.1. Droplet-Size Measurements

**Figure 6:**
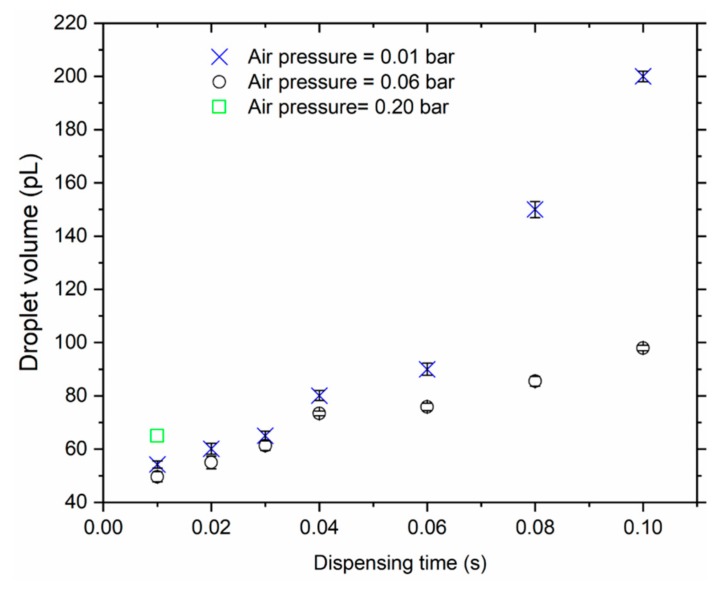
Volume of water droplets dispensed from a needle tip (of nominal internal diameter = 0.10 mm) on a cantilever sensor under various dispensing air pressures (0.06–0.20 bar) and pulse durations (0.01–0.10 s) at ambient conditions (*T* = 23.3 ± 0.5 °C; *rH* = 33 ± 4%).

By varying dispensing time and output pressure, various droplet sizes would be obtained. It was clearly observed (Figure 6) that, for the same dispensing pressure, the size of the water droplet increased with the pulse duration (*t*). By multiplying droplet mass with the water density (*ρ*_w_ = 1 gcm^−3^), the drop volume was obtained. Obviously, a small droplet size *V*_d_ = 49.7 ± 1.9 pL was measured for *t* = 10 ms for a dispensing pressure *p* = 60 mbar. Similarly, *V*_d_ = 98.1 ± 1.0 pL and 0.2 nL corresponded to *t* = 0.1 s for *p* = 0.06 bar and 0.2 bar dispensing air pressures, respectively.

Consequently, we used low output air pressure (60 mbar) and the shortest pulse duration (10 ms) to generate and realize all the droplets particularly aimed at sampling the particles from the solution. The quality factor *Q* before and after depositing the water droplet on the cantilever were, respectively, 1800 ± 250 and 153 ± 50. The latter shows a significant reduction in *Q*, which obviously depicts severe damping of the resonator when the water rests on the sensing surface. By assuming a spherical-shaped droplet of *V*_d_ ≈ 50 pL, a diameter ~45.6 μm was computed, which is much smaller than the width of the cantilever sensor (*w* ≈ 170 μm).

### 3.2. Evaporation Dynamics of Small Droplets on Microcantilever Surface

**Figure 7:**
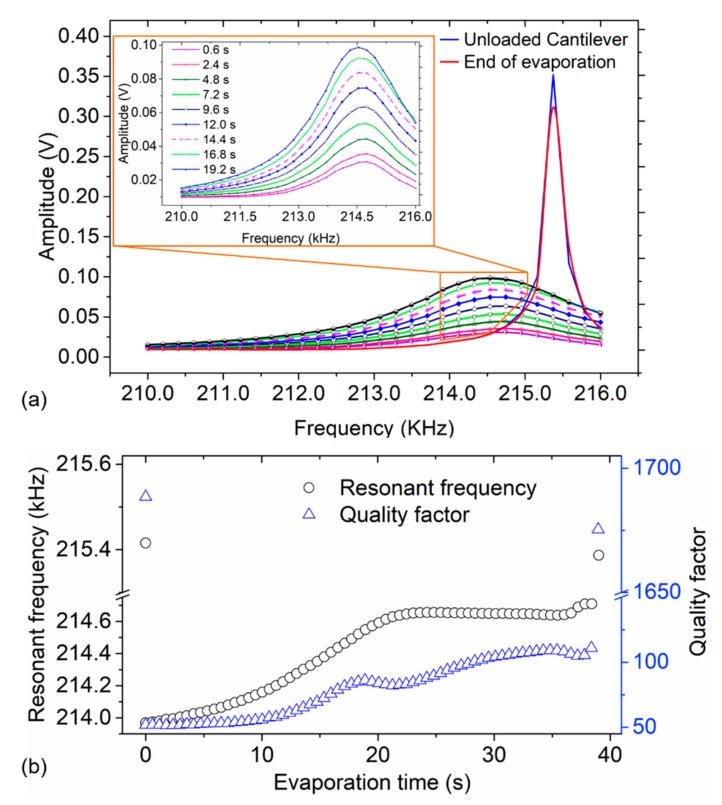
Offline monitoring of an evaporating water droplet (~50 pL) on a silicon-based piezoresistive microcantilever sensor under ambient atmospheric conditions (*T* = 24.4 °C, *rH* = 36%). (**a**) Frequency responses before and after droplet deposition, and during droplet evaporation at different time intervals. (**b**) Resonance frequency *f*_0_ and quality factor *Q* determined by fitting of a single harmonic oscillator (SHO) model to the curves in (a) over time during the evaporation period for a ~50 pL water droplet.

Water microdroplets on a cantilever sensor are bound to evaporate at ambient conditions. In our study, the loss of water droplet mass from the sensing surface was monitored by measuring the shift in resonant frequencies over time (as delineated in Figure 7a). This was particularly necessary to determine the droplet evaporation time *t*_ev_, and the minimum particle adsorption or waiting time prior to particle-mass measurement. Therefore, in determining *t*_ev_, the loss of droplet mass was initially monitored over time under ambient conditions through an offline resonant frequency measurement system (using a MFLI instrument). It should be noted (Figure 7a) that upon depositing the water droplet on the cantilever sensor, an immediate shift in resonant frequency was observed (i.e., *f*_0_ ~ 215.415 kHz shifted to *f*_r_ ~ 213.968 kHz), which corresponds to the added mass by the droplet. The total time taken for the cantilever to completely lose water from the surface and restore *f*_r_ to almost *f*_0_ (except for the shift induced by the mass of the attached particles) is equivalent to the drop evaporation time, *t*_ev_ = 39.0 ± 1.8 s.

It was clearly observed (in Figure 7a) that, once the water droplet was loaded onto the cantilever, the resonance frequency *f*_0_ rapidly shifts to a minimum whereupon the sensor momentarily stabilizes and recovers from this droplet-deposit impact. The cantilever then loses the water (~50 pL) into the ambient atmosphere within a span of about 39 s. Rapid evaporation and instantaneously loss of the water towards the last evaporation period was however observed. Subsequently, this culminates to a rapid shift of frequency (i.e., back to *f*_0_) and an abrupt rise in *Q* (i.e., from ~ 110 to about 1700). The corresponding trends and changes in resonant frequency and quality factor were evidently found to significantly change with the evaporation time (as depicted in Figure 7b). In Figure 7, an instantaneous change (increase) in the evaporation rate towards the winding phase is evidently demonstrated. Contrarily, Arcamone et al. [32] observed a decrease in the evaporation rate with time during the evaporation process. Further work is therefore necessary to interrogate the cause of the sudden and rapid water loss from the sensing surface particularly towards the last phase of an evaporating water droplet.

To theoretically evaluate the approximate evaporation time, we consider a spherical droplet cap of height *h*, contact radius *r*_c_, radius of the sphere forming the spherical cap *R*_S_, and contact angle *θ* (in radians), having a volume *V*_c_ given by [33]:

(5) $$V_{c}=\frac{\beta \pi }{3}R_{s}{}^{3},$$

where, $\beta =2-3\cos\theta +\cos^{3}\theta$ and $R_{s}=r_{c}/\sin\theta$. The relation of height of the spherical cap above the solid surface with *r*_c_ and *θ* is explicitly given in Equation (1) and can equivalently be related with *R*_s_ as: $h=R_{s}\left(1-\cos\theta \right)$. During droplet evaporation, the volume of the droplet on the solid surface at any time *t* can be computed as follows [33]:

(6) Vc2/3=(Vc,i2/3−23Kf(θ)·t)^(3/2),

where *V*_c,i_ denotes the initial droplet volume (at time *t* = 0 s) and the term 2/3*Kf*(*θ*)*t* represents the decrement in volume with time. The function $f\left(\theta \right)=-\cos\left(\frac{\theta }{2\ln\left(1-\cos\theta \right)}\right)\;$ is for the contact angle. For a constant contact-angle evaporation mode, we have f(*θ*) = f(*θ*_0_), where *θ*_0_ is the contact angle prior to evaporation (*θ*_0_ ~ 40° and *r*_c_ = 0.044 mm, i. e., f(*θ*_0_) = 0.263676, *β* = 0.1514, *R*_S_ = 0.070 mm and *V*_c,i_ = 49.7 pL). The parameter *K* ≈ 12.37467091*D*Δ*c*/(*ρ*_L_*β*^1/3^), in which the symbols *D,* Δ*c* and *ρ*_L_ denote the diffusivity (in cm^2^s^−1^) of the vapor molecules in the gas (i.e., air), the difference *c*_s_ − *c*_∞_ between the saturation concentration of water vapor at the sphere surface *c*_s_ (in gcm^−3^) and the ambient vapor concentration at infinite distance *c*_∞_ (in gcm^−3^) to the droplet, and the liquid density (in gcm^−3^), respectively. Assuming complete loss of water from the sensing surface, Equation (6) thus reduces to:

(7) $$t_{\mathrm{ev}}=\frac{{3V}_{c,i}{}^{2/3}}{{2Kf(\theta }_{0})}.$$

For a water droplet, *ρ*_l_ = *ρ*_w_ ≈ 1 gcm^−3^, *D* ≈ 0.282 cm^2^s^−1^ and *c*_s_ = *p*_v_*M*_w_/(*RT*) ~1.7081 × 10^−4^ gcm^−3^ given a molecular mass *M_w_* = 18.01528 gmol^−1^, the gas constant *R* = 8.314 J(mol·K) ^−1^ and the vapor pressure *p*_v_ in air of 22.2 mmHg (≅ 29.6 mbar) at a temperature *T* ≈ 24 °C [34] cm^2^s^−1^. Based on Equation (7), *V*_c,i_ = 49.7 pL and f(*θ*_0_) = 0.263676, we can therefore estimate the total time taken for the water droplet to completely vanish from the surface of a cantilever sensor to be about 37.14 s, which agrees very well with the experimental droplet evaporation time (*t*_ev_ ≈ 39 s).

## 4. Modeling Droplet-Particle Concentration: Wet and Dry Mass Approaches

### 4.1. Particle Adsorption on Cantilever and Resonant Frequency Responses

Wet mass and dry mass of the droplet can be measured immediately after depositing and evaporating the droplet, respectively. In both cases, a shift in resonant frequency is observed. Firstly, in the case of a wet drop, a shift in the resonant frequency can be observed from *f*_0_ (i.e., for a bare cantilever) to *f*_r1_ (cantilever + water + particles) as the droplet lands on the cantilever. Secondly, after a time lapse of *t*_ev_ the water is expected to evaporate and completely vanish from the sensing surface thereby shifting the resonant frequency *f*_r1_ to *f*_r2_ (i.e., cantilever + particles). The mass of the adsorbed particles can therefore most conveniently be computed (using Equation (4)) from the frequency shift of *f*_r1_ to *f*_r2_, i.e., Δ*f _=_ f*_r2_ − *f*_r1_. Similarly, the mass of the particle carrier fluid (i.e., water) can at the same time be determined from the frequency changes of *f*_r1_ (wet mass) to *f*_r2_ (dry mass), which nearly corresponds to the previously discussed evaporation trends (in Figure 7). In this study, however, since the evaporation time and droplet size had been determined previously, only *f*_r2_ and *f*_0_ were of essence.

**Figure 8:**
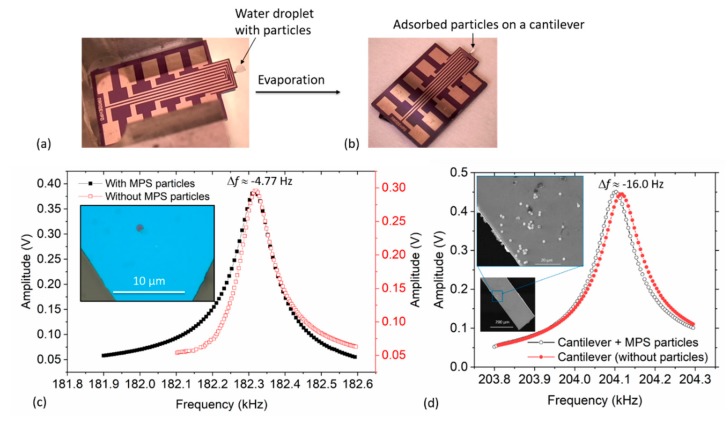
Optical images of: (**a**) particle-laden droplet on a cantilever, and (**b**) cantilever with adsorbed particles (after solvent evaporation). (**c**,**d**) Plots of resonant frequency measurements of a triangular and rectangular (normal) cantilever sensors with/without magnetic polystyrene (MPS) particles.

A typical water droplet containing MPS particles is presented in Figure 8a, from which the wet mass can most conveniently be obtained. As shown in Figure 8b, particles were adsorbed on the sensing surface after water evaporation thereby making it possible to determine the dry mass. In Figure 8c,d, for instance, we present typical frequency responses of microcantilevers with and without adsorbed magnetic particles. From these responses, the mass of the MPS beads was correspondingly determined from the observed frequency shift Δ*f* ≈ −4.77 Hz in accordance with Equation (1). These MPS beads were positioned at a point *x* ≈ 618.32 ± 44.20 µm (i.e., *U*(*x*_Δm_) ≈ −2.80) from the fixed end of the cantilever. It should be noted that, for *x ≈ L* = 1000 µm, the value of *U*(*x*_Δm_) ≈ −6.08. The measurements shown Figure 8c were achieved with *Q* = 2080 ± 430, for both the bare and loaded-state of the cantilever.

**Figure 9:**
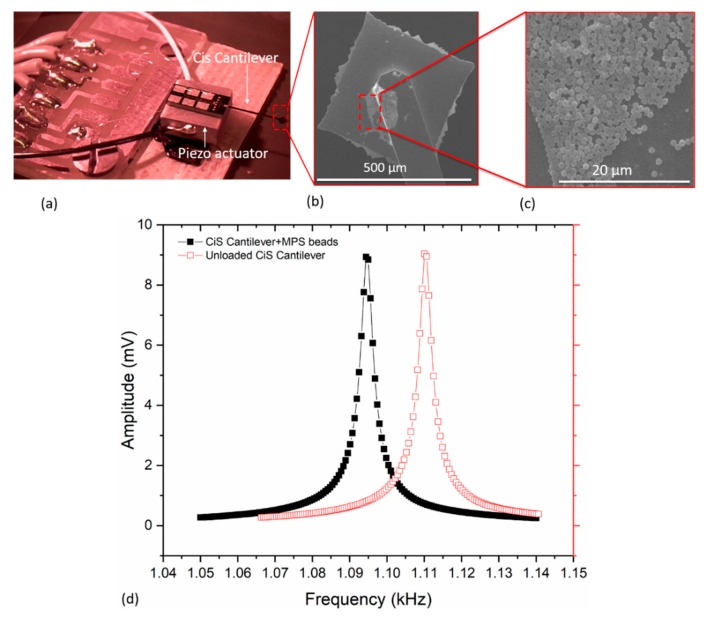
(**a**) Image of a wire-bonded CiS silicon cantilever sensor mounted on a thickness-extensional piezo-stack actuator; (**b**,**c**) SEM images of adsorbed MPS particles on the cantilever; and (**d**) measured resonance frequency responses before and after loading MPS beads on the sensor.

For comparability with commercial sensors, we similarly implemented and deposited MPS particles on a CiS cantilever sensor (CAN50-2-5, CiS Forschungsinstitut für Mikrosensorik GmbH, Germany, https://www.cismst.de/en/loesungen/mikrotastspitzen/). This silicon-based sensor was first cleaned, wire bonded (Figure 9a) and aligned (for particle deposition, cf. Section 2.4). The typical frequency response prior to and after adsorbing MPS beads on CiS cantilever is vividly shown in Figure 9d. The particles were adsorbed at a point *x* ≈ 4.80 mm from the fixed end, which apparently shifted the resonant frequency (*f*_0_ = 1.11 kHz) by −15.42 ± 0.04 Hz. This yielded a calculated particles mass of about 0.13 µg ± 0.3 ng, which is equivalent to about 36,473 ± 84 particles. This number was computed from the ratio of the adsorbate mass to single particle mass (which was estimated from the measured particle diameter of 1.83 ± 0.03 µm for MPS, assuming all particles are spherical in shape). From frequency response measurements (shown in Figure 9d), a quality factor *Q* of about 379 ± 3 was realized, depicting a fairly stable operating environment. This cantilever was externally excited in its fundamental out-of-plane mode with a piezo actuator, and the connections to and from the MFLI amplifier were done (as illustrated previously in Figure 4).

**Figure 10:**
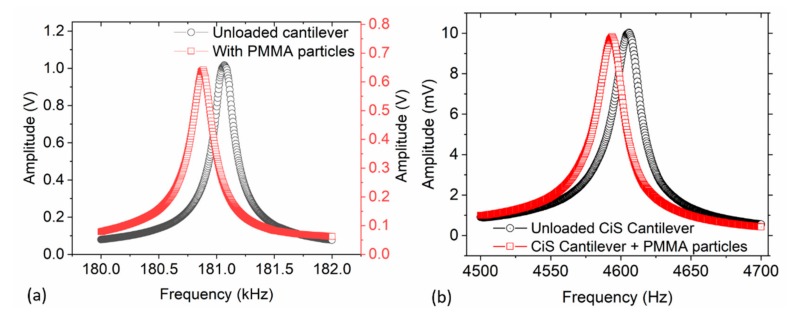
Frequency measurements depicting responses with and without PMMA particles using: (**a**) a triangular (in-house) cantilever; and (**b**) CiS commercial sensor.

In Figure 10, we further present frequency responses due to adsorbed PMMA particles on a triangular cantilever (in-house) and a CiS cantilever (CAN30-1-2, *m*_0_ ≈ 20 µg, https://www.cismst.de/en/loesungen/mikrotastspitzen/). The observed resonant frequency shift in Figure 10a was about −181.1 Hz (with *Q* ≈ 1700) while in Figure 10b Δ*f* ≈ −11.8 Hz (with *Q* ≈ 285), corresponding to approximately 5.73 ng (i.e., 1449 particles) and 4.07 ng (i.e., 1036 particles), respectively. The number of calculated PMMA particles (based on point-mass condition) from other measurements ranged from about 56 to 6169. The assumed particle density and measured diameter were 1.18 gcm^−3^ and 1.90 ± 0.05 µm, respectively. A comparison of the number of particles estimates (both for MPS and PMMA) based on resonant frequency responses and particle counts (from SEM) is presented in the next section.

### 4.2. Microscopic Particle count and Analysis

Particles dispersed in the liquid (droplet) are transported by the flow and deposited or assembled on a solid surface as the solvent evaporates. Depending on solid-surface properties and particle concentration, particles may form monolayers, multilayers, or both. In the latter case, for instance, a monolayer segment might be formed at the center while a progressive accumulation of particles leads to the formation of cluster-ring-shaped patterns after drying is complete, which often results in multilayers [35,36]. In the present study, particles were deposited and adsorbed on a hydrophilic Si surface. Generally, such a surface is highly adhesive and allows convective flow of fluid carrier thereby leading to the self-assembly of particles upon solvent evaporation.

**Figure 11:**
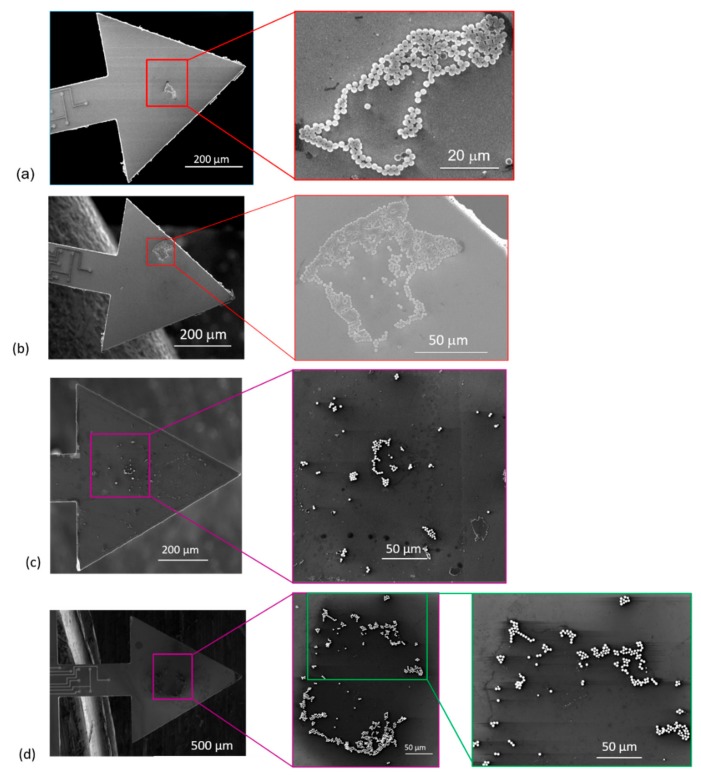
SEM images of adsorbed particles on cantilevers depicting monolayer particle distribution profiles: (**a**,**b**) sensors consisting of adsorbed MPS particles; and (**c**,**d**) sensors adsorbed with PMMA particles. Monolayer particles in (**c**,**d**) are positioned along the axisymmetric axis, whereas in (**a**) the particles were adsorbed nearly at center of the equilateral triangular surface. In (**b**), however, some MPS particles were adsorbed very close to the edge of the cantilever.

Initially, MPS particle count estimate (from SEM micrographs) for single droplets deposited on Si bulk substrates was found to be 104,470 ± 420. However, by tuning the particle concentration and droplet size, a small particle assembly was realized on the cantilever surface (as shown in Figure 11). Prior to SEM particle-imaging, the resonant frequency response due to the attached particles (on the cantilever) was measured and the corresponding mass was determined thereof. The same sample was then mounted in the SEM and particle-imaging was performed. Subsequently, the number of particles and adsorbate position (*x*) were estimated in ImageJ [24].

**Figure 12:**
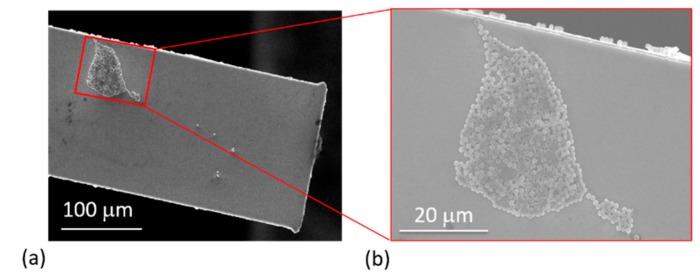
SEM images of MPS particles deposited on a rectangular cantilever (**a**); and the magnified view depicting a multilayered particles arrangement (**b**).

In every measurement cycle, the cantilever was cleaned as described above. Typically, a monolayer particles distribution of the adsorbed on the sensors was observed (Figure 11). For MPS, SEM particle-counts of about 19 ± 2 (Figure 8c, inset), 60 ± 6 (Figure 8d, inset) and 203 ± 8 (Figure 11a) were determined, whereas, for PMMA, the counted particles were 47 ± 6 (least count), 123 ± 8 (Figure 11c) to 671 ± 36 (Figure 11d). The particle agglomeration patterns for both MPS and PMMA (as depicted in the SEM images shown in Figure 11) evidently show a similar trend. In some other instances, however, a monolayer plus cluster(s) of non-uniform multilayers was observed (Figure 12). In such a case, only particles on the top layer could be counted with certainty to give a minimum number of the adsorbate (e.g., >300).

**Figure 13:**
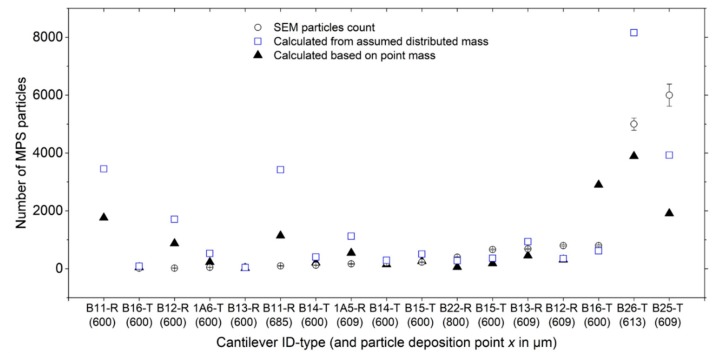
Comparison of the number of MPS particles calculated from resonant-frequency shifts (considering point-mass and distributed-mass conditions) with the particle-count results from SEM imaging. The last letter in the cantilever ID-type, i.e., T and R, denote triangular and rectangular cantilever types, respectively.

In Figure 13, we further present a summary of MPS particle counts (from SEM imaging) and compare the same with the number of particles calculated from resonant frequency measurements. For the same adsorbate, the number of particles due to both point-mass (Equation (4)) and distributed-mass ($\Delta m\;=-{2m}_{\mathrm{eff}}\Delta f/f_{0}$) conditions was calculated. From both mass distributions, some disparities in the number of particles were observed. Nonetheless, the number of particles due to point mass was fairly comparable with the particles-count results from the SEM. Generally, the observed disparities in the number of particle (from the two approaches) were attributed to: some drift and inconsistencies in positioning of the dispensing tip (and therefore the droplet on the sensor), and formation of multilayers. The former is a consequence of tip misalignment and may lead to spilling or adsorbing of particles along the edges of the beam (as depicted in Figure 12b), whereas the latter introduced uncertainties in particle-count estimations.

### 4.3. Accuracy of Mass Measurements

In sensing and determining the mass due to frequency shift induced by adsorbed particles or otherwise, knowledge of the measurement environment (especially relative humidity r*H* and temperature *T*) plays a critical role. In our study, these parameters (r*H* and *T*) were adequately monitored in every measurement cycle and found to be relatively stable. It is however worth noting that some little water from ambient atmosphere would possibly be adsorbed on the sensing surface courtesy of the hydrophilicity of our sensing surface and magnetic particles. In the current study, because of stable r*H* environment, we considered its contribution to the calculated mass to be negligible.

The position of particles on the sensing surface also affects the frequency response of the sensor due to the added mass (Equation (4)). Depositing particles away from the intended axisymmetric or eccentric target points would therefore clearly influence such output. In our work, such observations were evidently shown (e.g., Figure 11b and Figure 12b) and were mainly associated with the misalignment of the dispensing tip and/or relative change of the wettability of the sensing surface. The latter is possibly due to the interplay of wet cleaning(s) of the sensors and natural oxidation of sensing surface, parameters which can potentially tune the already hydrophilic sensing surface. If this surface is rendered more hydrophilic, the droplet may rapidly spread on the sensing surface and overflow unto the edges. Contrarily, spreading of liquid surface on less hydrophilic surface is slow and the droplet (hence, particles) can be fairly localized. Further work would therefore be necessary to enhance positioning with a better uncertainty. Amongst such considerations include coating the edges of the cantilever with a hydrophobic substance such as trimethoxy (octadecyl) silane (OTMS). This chemical compound would substantially help in confining the droplet within the sensing area and, hence, eliminate or minimize possible spillages. Basically, OTMS is compatible and forms strong covalent bonds with silicon oxide surfaces [37]. For our microcantilevers, such coating procedure would be integrated and performed in the last stages of the fabrication process.

**Figure 14:**
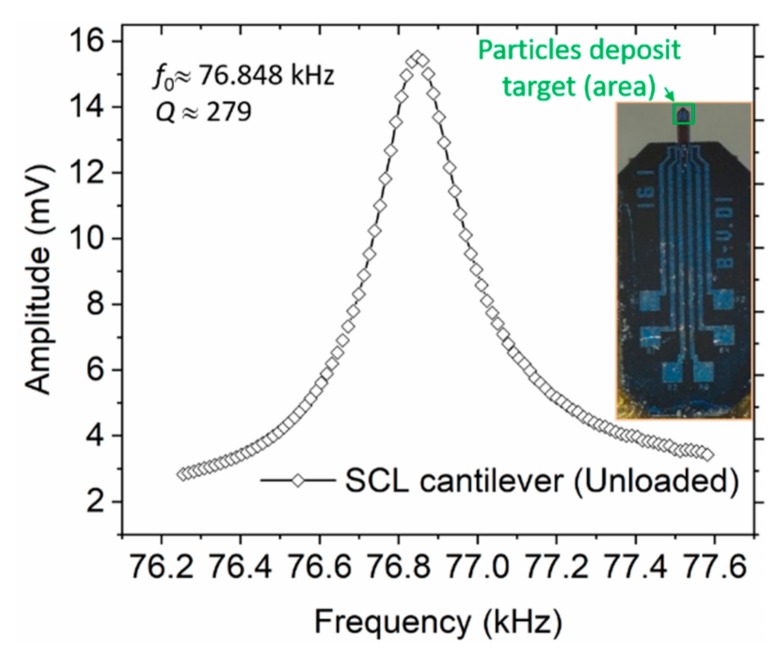
Measured frequency of a SCL tipless cantilever sensor. Inset is the photo of the sensor illustrating the possible particle-adsorption and -sensing area.

Moreover, to enhance mass sensitivity towards detectability of single liquid-borne particles, scaling down of our micro-sized sensors is further intended. In our recent works, efforts towards this course have successfully culminated to a self-readable resonant cantilever of a mass of *m*_0_ = 2 −5 ng and a sensitivity of about 7.8 fg/Hz meant for the detection of airborne nanoparticles [38,39]. Nonetheless, in Figure 14, we illustrate our intended integration of tipless cantilever chips (PRSA-L300-F80-TL, from SCL-Sensor. Tech. Fabrication GmbH, https://www.sclsensortech.com/self-sensing-cantilevers-tipless/) with *m*_0_ ~ 0.5 µg (a factor of 30 and 50 lower than our rectangular and triangular cantilevers, respectively) into our measurement setup. These sensors offer the possibility of sensing micro particles with increased sensitivity. Calibration of these cantilever mass sensors would also favorably be considered to give minimal bias between the resonant measurement and SEM-particle count estimations.

## 5. Conclusions

In this work, we sampled and adsorbed liquid dispersed particles on our in-house fabricated silicon-based piezoresistive cantilever sensors and further successfully tested the same approach on CiS commercial cantilever sensors. The mass sensitivities of our sensors ranged from about 27 Hz/ng to 14.73 Hz/ng for rectangular and triangular free-ended cantilever, respectively. With these cantilever sensors, we determined the masses of attached water droplets and particles through resonant frequency responses and compared the findings thereof with SEM particle count analysis. In our measurement process, droplet dispensing parameters were optimized to realize a small water droplet volume *V*_d_ = 49.7 ± 1.9 pL which perfectly fitted on the sensing surface under consideration. Furthermore, water droplet evaporation trends were also investigated to ascertain minimum waiting time prior to particle-mass measurements. In general, a total evaporation time *t*_ev_ ≈ 39 s was determined and found to compare well with the theoretical estimation (37.14 s). Particle adsorption on hydrophilic Si bulk substrates and piezoresistive microcantilevers is also presented, followed by a determination of their mass and number based on both resonant frequency shift measurements and SEM image analysis for particle counting. Monolayer particles assembly on the sensor with a lowest particle count of about 19 and 47 for MPS and PMMA particles were, respectively, realized with a high-quality factor *Q* ≈ 1900, which nearly corresponds to *Q* of a bare cantilever.
